# Estimating the price at which hepatitis C treatment with direct-acting antivirals would be cost-saving in Japan

**DOI:** 10.1038/s41598-020-60986-4

**Published:** 2020-03-05

**Authors:** Yueran Zhuo, Tomoyuki Hayashi, Qiushi Chen, Rakesh Aggarwal, Yvan Hutin, Jagpreet Chhatwal

**Affiliations:** 1 Massachusetts General Hospital Institute for Technology Assessment, Boston, MA USA; 2 World Health Organization, Geneva, Switzerland; 3 Department of Gastroenterology, Kanazawa University and WHO Collaborating Center for Chronic Hepatitis and Liver Cancer, Kanazawa, Ishikawa Japan; 4 The Harold and Inge Marcus Department of Industrial and Manufacturing Engineering, Pennsylvania State University, University Park, PA USA; 5 Jawaharlal Institute of Postgraduate Medical Education & Research, Puducherry, India; 6 Harvard Medical School, Boston, MA USA

**Keywords:** Health care economics, Health policy

## Abstract

In Japan, 1.5–2 million people are chronically infected with hepatitis C virus (HCV) infection. New direct-acting antiviral agents (DAA) offer an unprecedented opportunity to cure HCV. While the price of HCV treatment decreased recently in most countries, it remains one of the highest in Japan. Our objective was to evaluate the cost-effectiveness of HCV treatment in patients of different age groups and to estimate the price at which DAAs become cost-saving in Japan. A previously developed microsimulation model was adapted to the Japanese population and updated with Japan-specific health utilities and costs. Our model showed that compared with no treatment, the incremental cost-effectiveness ratio (ICER) of DAAs at a price USD 41,046 per treatment was USD 9,080 per quality-adjusted life year (QALY) gained in 60-year-old patients. HCV treatment became cost-effective after 9 years of starting treatment. However, if the price of DAAs is reduced by 55–85% (USD 6,730 to 17,720), HCV treatment would be cost-saving within a 5 to 20-year time horizon, which should serve to increase the uptake of DAA-based HCV treatment. The payers of health care in Japan could examine ways to procure DAAs at a price where they would be cost-saving.

## Introduction

Hepatitis C virus (HCV) infection affects 71 million people globally^1^. If untreated, HCV infection can lead to cirrhosis, hepatocellular carcinoma (HCC) and liver-related death. Directly-acting antivirals (DAAs), a class of HCV treatment available since 2014, offer an unprecedented opportunity to reduce the disease burden. The cure rate (defined by sustained virologic response [SVR])] with these medicines exceeds 95%, irrespective of patients’ prior treatment history, HCV genotype, or fibrosis stage^2^.

In Japan, the prevalence of HCV infection is 0.6–0.9%; one of the highest among high income countries, with approximately 1.5–2 million chronically infected people^3,4^. According to the 17th nationwide follow-up survey of primary liver cancer in Japan, HCV infection causes 70% of all liver-related deaths^5^. In 2014, the Japan Ministry of Health, Labor and Welfare approved DAAs for HCV treatment, and the Japan Society of Hepatology guidelines recommended using them for treating HCV infection ever since^6,7^. Despite their availability in Japan, treatment uptake rates of DAAs have remained low, which is due to the low proportion of infected persons diagnosed resulted by the lack of testing in the population^8^.

Because HCV infection is a slow progressive disease, benefits of treating HCV infection are accrued several years after the treatment in terms of prevention of liver complications such as end-stage liver disease, and hepatocellular carcinoma. Therefore, the upfront cost of treating a large population can constrain the payers’ limited budget. While in many high-income countries, the prices of DAAs have come down substantially (e.g., USD 25,000 in the United States and USD 12,439 in the United Kingdom), the price of a 12-week regimen of DAAs in Japan ranges between USD 32,480–42,060 in 2019. Furthermore, it is shown that DAAs are cost-saving (i.e., increase QALY but reduce the total cost associated with HCV) in countries such as United States^9^ and United Kingdom^10^, but none of the published studies has evaluated if and at what price HCV treatment becomes cost-saving in Japan^11–16^. To fill this gap, the objective of this study was to evaluate the feasibility of HCV treatment with DAAs becoming cost-saving in Japan.

## Methods

### Model overview

We utilized a previously developed individual-level state-transition model, the *Markov-based Analyses of Treatments for Chronic Hepatitis C* (*MATCH)*^17,18^, to evaluate the cost-effectiveness of HCV treatment in Japanese population. The natural history of our MATCH model has been validated with previously published cost-effectiveness studies^19–21^ and a multicenter follow-up study in the United States of patients with advanced fibrosis. The model was developed using C++ computer programming language, and was designed to follow the principles recommended by a reference group convened by the World Health Organization (WHO) on economic analyses in the field of viral hepatitis^22^.

### Characteristics of base case population

We simulated a total of 150 unique HCV-patient profiles based on five age categories (30, 40, 50, 60 and 70 years), three HCV genotypes that are prevalent in Japan (G1, G2, G4), patient’s sex (male or female) and METAVIR fibrosis score (no fibrosis [F0], portal fibrosis without septa [F1], portal fibrosis with few septa [F2], numerous septa without fibrosis [F3], or cirrhosis [F4])^23^. The distributions of these HCV-infected patient profiles were estimated based on available data (Supplementary Table S1). The model did not include patients with HIV or hepatitis B virus co-infection as well as special groups at higher risk of HCV reinfection, such as those with hemodialysis, thalassemia, haemophilia or injection drug use. All patients were considered treatment-naïve because the current percentage of treatment-experienced patients in Japan is small.

### Treatment regimens and efficacy

We simulated two strategies: no treatment and treatment with available DAAs. The DAA treatment regimens used were determined by individual patient’s HCV genotype and METAVIR fibrosis stage. The treatment efficacy in various scenarios was based on the SVR rate reported in clinical trials of DAAs, and uncertainty in SVR rates was incorporated in sensitivity analysis. Regimen-specific treatment discontinuation rates were also incorporated in the model. Data about the treatment regimens were obtained from recent clinical trials of DAAs in treatment-naïve patients^24–26^, and are provided in Supplementary Table S2.

### Natural history of HCV infection

**Figure 1:**
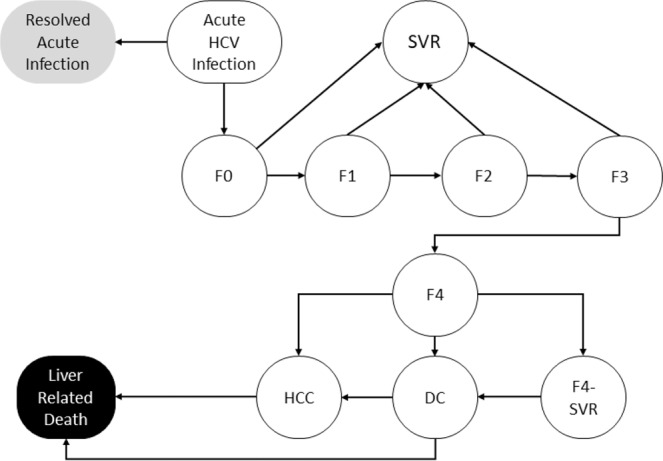
State-transition model of the natural history of HCV. At any given time, a patient is represented by one of the health states, which are shown by squares. Arrows between states represent possible transitions based on annual probabilities. Patients whose disease is successfully treated transition to the sustained virological response (SVR) state. Patients who achieve SVR from F0 to F3 states are assumed to be cured; however, patients in an F4 state who are successfully treated transition to an F4-SVR state and may develop further complications albeit at a slower rate than the untreated F4 state. Patients in HCC, and DC states have a higher mortality rate than the general population. All other patients have the same risk of death as the general population. The probability of death from other causes exists in every state, but deaths from other causes are not shown in the scheme above. According to the Meta-analysis of Histologic Data in Viral Hepatitis (METAVIR) scoring system, F0 indicates no fibrosis of the liver, F1 indicates portal fibrosis without septa, F2 indicates portal fibrosis with few septa, F3 indicates many septa without cirrhosis, and F4 indicates cirrhosis.. *Abbreviations*: DC, decompensated cirrhosis; HCC, hepatocellular carcinoma; HCV, hepatitis C virus; SVR, sustained virologic response.

As time progresses, an HCV-infected patient could transition between several Markov health states in the model (Fig. 1**)**. The cycle length of our model was set as one week. Each patient would start in one of the five METAVIR liver fibrosis states (F0–F4). At the end of each cycle, F0–F3 patients could move to a higher fibrosis stage or stay in the same state. F4 patients could progress into decompensated cirrhosis and/or HCC, or could die because of liver-related mortality. Patients in all five liver fibrosis states (F0-F4) could achieve SVR following treatment, but only those in F0-F3 were assumed to be cured. Patients in F4 state who achieved SVR could still progress to more advanced states, although at a slower rate (Table 1)^27^. Patients who failed to achieve SVR or discontinued treatment continued to progress over time. All states were subject to a background age-specific mortality, which was based on Japan life table^28^.

**Table 1:** Annual transition probabilities, healthcare costs and quality of life weights for different Markov states.

Input	Base case	Values for sensitivity analysis			
		Range	Distribution	Parameter 1^b^	Parameter 2^c^
**Transition** ***probabilities (annual)***^a^					
F0 to F1^29^	0.117	0.104–0.130	Beta	274.98	2,075.30
F1 to F2^29^	0.085	0.075–0.096	Beta	210.06	2,261.18
F2 to F3^29^	0.120	0.109–0.133	Beta	288.05	2,112.38
F3 to F4^29^	0.116	0.104–0.129	Beta	270.61	2,062.22
F4 to DC^30^	0.039	0.010–0.079	Beta	3.51	86.48
F4 to HCC^30^	0.014	0.010–0.079	Beta	0.18	12.38
Post F4-SVR to DC^27^	0.008	0.002–0.036	Beta	0.31	38.58
Post F4-SVR to HCC^27^	0.005	0.002–0.013	Beta	1.49	297.13
DC to HCC^46^	0.068	0.030–0.083	Beta	73.58	1008.49
DC (first year) to death from liver disease^46^	0.182	0.065–0.190	Beta	1626.40	7309.88
DC (subsequent year) to death from liver disease^46^	0.112	0.065–0.190	Beta	7.03	55.77
HCC to death from liver disease^30^	0.427	0.330–0.860	Beta	2.14	2.87
***Health state costs (annual in JPY)***					
F0-F3^32–34,47,48^	403 (¥44,213)^d^	0.5–12.3 fold	Gamma	313	0.777
Compensated cirrhosis^32–34,48^	1301 (¥142,733)	0.5–3.7 fold	Gamma	970	0.746
Decompensated Cirrhosis^32–34,48^	6,921 (¥759,303)	0.5–2.0 fold	Gamma	5008	0.723
Hepatocellular Cancer^32–34,48^	15,618 (¥1,713,451)	0.5–2.0 fold	Gamma	11214	0.718
**Testing cost (JPY)**^e^					
Pre-treatment (diagnosis)	71 (¥7,789)	0.5–2.0 fold	Gamma	17.11	4.14
Post-treatment	40 (¥4,388)	0.5–2.0 fold	Gamma	17.11	2.33
***Health state quality-of-life weights***					
Anemia multiplier^*a*^^49^	0.83	0.66–0.97	Beta	22.95	4.70
F0–F3^15,35^	0.82	0.58–0.99	Beta	36.86	8.03
Compensated cirrhosis^15,35^	0.74	0.54–0.99	Beta	45.44	16.21
DC^15,35^	0.67	0.45–0.99	Beta	50.89	24.95
HCC^15,35^	0.56	0.77–0.99	Beta	85	65.17
Post-SVR^13^	0.96	0.92–1.00	Beta	25.47	1.06

Abbreviations: SVR, sustained virologic response; F0–F4, METAVIR fibrosis score; DC, decompensated cirrhosis; HCC, hepatocellular carcinoma; F4-SVR. Post-SVR state of treated cirrhotic patient.

YEN (¥), Japanese Yen.

^a^Same value also reported being used in another study in India population^18^.

^b^Parameter 1 corresponds to *α* parameter for beta distribution and *k* (shape) parameter for gamma distribution.

^c^Parameter 2 corresponds to *β* parameter for beta distribution and *θ* (scale) parameter for gamma distribution.

^d^conversion rate: 1USD = 109.71JPY.

^e^Expert opinions from Tatsuya Yamashita, Department of Gastroenterology, Kanazawa University, Kanazawa, Ishikawa, Japan. For patients who experienced anemia during treatment, quality of life was multiplied by this factor.

Data on the fibrosis progression rates from F0 to F4 were obtained from a meta-regression analysis^29^, and progression rate from cirrhosis to decompensated cirrhosis and HCC were modeled based on retrospective follow-up studies of HCV-infected patients^30^. Patients with decompensated cirrhosis or HCC had a higher liver-related mortality than those in early stages of HCV infection^31^. As the number of liver transplants performed in Japan is very small compared to the total number of HCV-infected persons, we did not consider liver transplant as part of the HCV disease progression or treatment in this model^13^.

### Medical costs

We considered three types of HCV-related costs in the model: the costs for pre-treatment diagnosis and post-treatment monitoring testing, the DAA treatment costs and the disease management costs for each health state over a person’s life-time. All costs are presented in U.S. dollars (USD). For estimation of the cost values, we adopted an ingredients approach where two elements were considered: the unit *price* of commodities or services used ($p$) and the *quantity* of the required commodities or service ($q$). The total cost for a sequela is the product of these two elements as $p\times q=Cost$. For the cost values presented in this study, we collected the relevant inpatient charges and medical fees from the database of the Ministry of Health, Labor and Welfare in Japan^32–34^, and sought expert opinions for the quantities of prescription and services required. We adopted the current price of ledipasvir 90 mg/sofosbuvir 400 mg for the base case treatment cost in the model, which amounts to a cost of 41,046 USD per treatment. The cost of pre-treatment testing for fibrosis staging and HCV genotyping was taken as USD 71 (7,789 JPY), and the testing cost for investigations during and after treatment was taken as USD 40 (4,388 JPY). Data sources and the cost values are summarized in Table 1.

### Quality of life weights

We assigned quality-of-life (QoL) weights to each health state in the model. These weights were used to determine quality-adjusted life years (QALYs) and were derived from previously-published studies^21,35,36^ (Table 1). The QoL of patients in F0-F3 who had achieved SVR was assumed to be equivalent to that of the general, non-HCV-infected population; however, for those patients who achieved SVR at F4 stage and above, the QoL of the corresponding advanced liver disease states was used.

### Cost-Effectiveness analysis

We simulated the clinical course of HCV-infected patients in Japan with and without DAA treatment, respectively. For each patient profile, we simulated a cohort of size 10,000 to project the total expected life-years, QALYs and costs during the lifetime horizon. All QALYs and costs were discounted at 2% per year^37^. Based on the projected costs and QALYs, we estimated the incremental cost-effectiveness ratio (ICER; USD per QALY) of DAA-based treatment in comparison with no treatment. Our analysis was conducted from healthcare payer’s perspective. For each patient profile under each horizon, we also projected the cumulative incidence of decompensated cirrhosis and HCC, and liver-related deaths.

To evaluate the impact of DAA medicine price on outcomes, we performed threshold analysis on DAA medicine price and examined the ICERs under different prices. Specifically, we calculated the price at which DAA treatment becomes cost-saving at the end of the next 5 years, 10 years and 20 years, respectively.

### Sensitivity analyses

We performed one-way sensitivity analyses to evaluate the effects of several model inputs on the cost-effectiveness of HCV treatment. These included state transition probabilities, QoL weights, medical and disease management costs, and discount rate. The ranges of disease management costs cover the reported values in similar studies^11,13,15^. We also performed a probabilistic sensitivity analysis using 10,000 first-order and 5,000 second-order samples by simultaneously varying all key model inputs using the recommended statistical distributions. To examine the effect of DAA prices more specifically, we conducted these sensitivity analyses using DAA prices identified in the threshold analysis. Life-time horizon was used for the sensitivity analysis. The range of all model inputs used for sensitivity analyses are in Table 1.

## Results

### Base case cost-effectiveness analysis

Compared with no treatment, the use of DAAs in Japanese patients with HCV infection at a mean age of 60 increased the overall life expectancy by 3.16 years and discounted QALYs by 2.67 years. The no-treatment strategy had a lifetime cost of USD 23,206 per person infected (all spent on managing consequences of HCV infection); the DAA scenario resulted in higher lifetime cost of USD 47,431 per person infected, with 91% spent on DAA treatment, and smaller amounts on testing (1%) and HCV disease management (8%); resulting in an ICER of USD 9,080 per QALY gained. In the baseline scenario, DAA treatment was cost-effective at a willingness-to-pay (WTP) threshold of USD 45,960 (JPY ¥5,000,000)^38^, but not cost saving.

DAA treatment was more cost-effective in younger patients – ICER was USD 1,998/QALY for age 30 versus USD 24,085 per QALY gained for age 70 (Table 2). However, HCV treatment was not cost-saving for any of the age groups at the 2019 price of DAA medicines.

**Table 2:** Cost-effectiveness of direct-acting antivirals treatment (versus no treatment) in Japan, by age group, 2018.

Patient age (years)	Life Years (LYs)			Quality-adjusted Life Years (Discounted)			Total Life-time Cost (Discounted USD)		ICER (USD/QALY)
	No treatment	With DAA-based treatment	Increase in LYs	No treatment	With DAA-based treatment	Increase in QALYs	No treatment	With DAA-based treatment	
*30*	26.89	42.84	15.95	12.80	20.61	7.81	35,106	50,703	1,998
*40*	24.66	35.44	10.79	11.81	17.84	6.03	32,759	49,857	2,833
*50*	21.52	27.92	6.40	10.49	14.76	4.27	29,010	48,728	4,620
*60*	17.16	20.33	3.16	8.62	11.29	2.67	23,206	47,431	9,080
*70*	11.49	12.47	0.99	5.99	7.27	1.28	15,066	45,809	24,085

Abbreviations: *DAA*, direct-acting antivirals; *ICER*, incremental cost-effectiveness ratio; *LYs*, life years; *QALY*, quality-adjusted life year.

Comparing to the willingness-to-pay threshold of USD 45,960 (JPY 5,000,000) in Japan, the DAA-based HCV treatment is cost-effective patients in all age groups from 30-year-old to 70-year-old. However, even for the youngest age group of HCV patients, DAAs are still not cost-saving. To become cost-saving in foreseeable years after the treatment, the prices of DAA medicines must be reduced substantially. ICER value is lower for patients with younger ages, i.e., DAA-based HCV treatment is more cost-effective for younger patients.

Treating per 10,000 persons without cirrhosis using DAAs could prevent 2,773 cases of decompensated cirrhosis, 1,611 cases of HCC, and 2,994 liver-related deaths, compared with the no-treatment scenario. In 10,000 cirrhotic patients, treatment could prevent 3,172 cases of decompensated cirrhosis, 1,795 cases of HCC and 3,520 liver-related deaths (Supplementary Fig. S1).

### Cost-effectiveness over time and impact of price of DAA regimens

**Figure 2:**
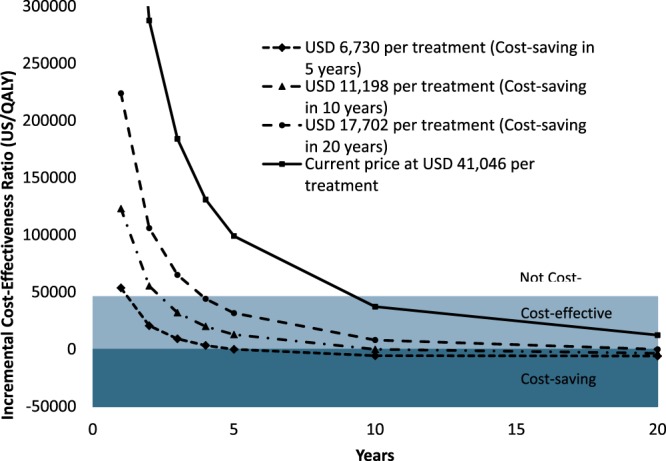
Cost-effectiveness of treatment based on direct-acting antiviral (DAAs) of persons with hepatitis C virus infection over time. At the current cost of USD 41,046 per treatment (solid line), DAAs are not cost-saving, but become cost-effective (cross the cross-effectiveness threshold [USD 45,960 per QALY]) at the end of 10 years. DAAs become cost-saving (i.e. ICER falls below zero) in 20 years if the cost of medicines is reduced to USD 17,702 per treatment in 10 years if the cost of medicines is reduced to USD 11,198 per treatment and in 5 years if the cost of medicines is reduced to USD 6,730 per treatment.

At the 2019 cost of DAAs, USD 41,046 per treatment, HCV treatment became *cost-effective* after 9 years of starting treatment in HCV patients at age 60 (Fig. 2). However, at this cost, DAAs are *not cost-saving* in Japan.

HCV treatment could become *cost-saving* within 20 years if the DAA cost was reduces to USD 17,702 per treatment (57% reduction compared with the current listed price); further, it could become cost-saving within 10 years if the cost was reduced to USD 11,198 per treatment (73% reduction), and within 5 years if the cost was reduced to USD 6,730 per treatment (84% reduction) (Fig. 2).These four different DAA costs were used for the cost-effectiveness and sensitivity analyses that follows.

### Cost-effectiveness by age and fibrosis stage

**Figure 3:**
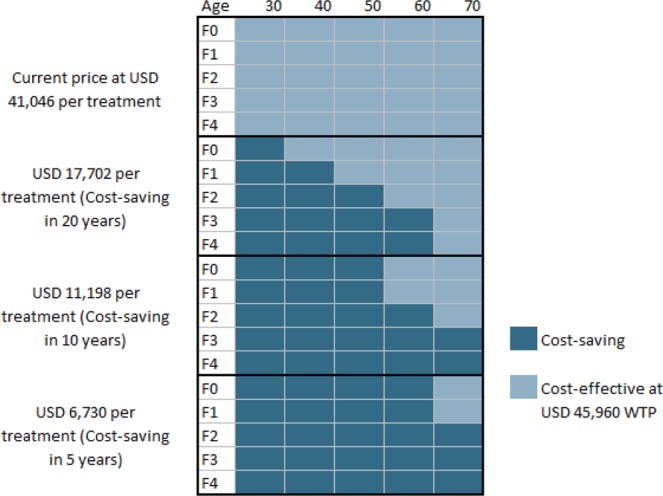
Under the 2019 price, DAA treatment is not cost-saving for patients irrespective of their age and fibrosis stage. When DAA cost is reduced to USD 17,702 per treatment, it would become cost-saving for younger patients with higher fibrosis stages. Further reducing the DAA cost to USD 11,198 would make HCV treatment cost-saving in more subgroups. If DAA cost were further reduced to USD 6,730 per treatment (89% reduction), DAA would become cost-saving for all age groups from and fibrosis stages in an average duration of 5 years with the only exception of F0-F1 patients of age 70.

With the 2019 baseline cost, DAA treatment was not cost-saving for any of the patient groups, based on the age and fibrosis stage considered (Fig. 3). However, if DAA cost were reduced to USD 16,104 per treatment, treatment would become cost-saving in younger patients and/or in those at early fibrosis stage. Further reducing the DAA cost to USD 11,198 would make HCV treatment cost-saving in more subgroups. If DAA costs were further reduced to USD 6,730 per treatment (84% reduction), HCV treatment would become cost-saving for all age groups and fibrosis stages in an average duration of 5 years.

### Sensitivity analysis

**Figure 4:**
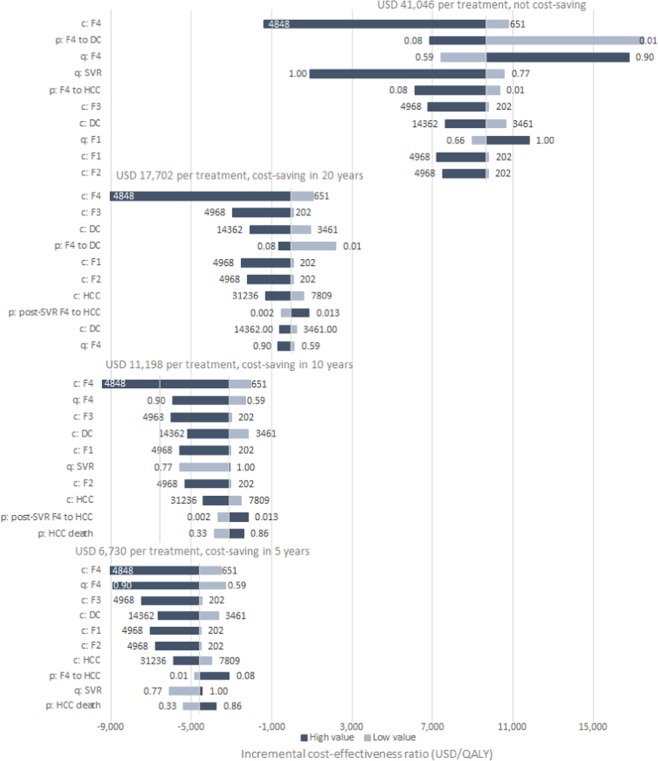
Tornado diagram for one-way sensitivity analysis under four different DAA prices of incremental cost-effectiveness ratio using per additional quality-adjusted life-year. Horizontal bars show the variation in incremental cost-effectiveness ratio (ICER; in USD/QALY) with variation in the value of the parameter. In the parameter names, the prefix ‘c’ represents cost of a health-state, ‘q’ the quality-of-life weight and ‘p’ the transition probability from one state to the other. Values of ICER below 0 indicate that the treatment is cost-saving. Abbreviations: QALY = quality-adjusted life-year.

Figure 4 shows the 10 most sensitive model parameters to ICER results under four DAA costs respectively. For the base case, i.e., using the current DAA cost in Japan, the most influential parameters included cost of managing HCV disease through fibrosis stage F1 to decompensated cirrhosis, disease progression rate in patients with cirrhosis (with or without SVR), and quality of life utility value of cirrhosis and post-SVR status. Many of the above variables remained influential when DAA treatment cost is reduced to lower values. However, at lower DAA cost, the ICER also became sensitive to the disease progression rate in patients with cirrhosis with SVR, and cost of managing HCC.

The probabilistic sensitivity analysis indicated that DAA treatment was cost-effective but not cost-saving under any circumstance at the 2019 DAA cost. However, under the hypothesis by which the cost was reduced to USD 17,702 per treatment, DAA would become cost-saving in 20 years with the likelihood of 99.03%. If the cost were further reduced to USD 11,198 and USD 6,730, DAA would become cost-saving in 10 years and 5 years, and with 99.72% and 99.93% likelihood, respectively.

## Discussion

The availability of DAAs has profoundly changed the HCV treatment paradigm, bringing hope for elimination of HCV as a public health threat by 2030^39^. Because hepatitis C treatment is expensive in Japan, providing treatment to a large population requires a substantial budget. In such a situation, showing that the treatment can result in cost savings in short time horizon (e.g., 5 years) can encourage the policy makers push for more aggressive treatment plans for the mass HCV infected population, thus increase the HCV uptake rate. Our study showed that HCV treatment in Japan, although cost-effective, was not cost-saving in any of the age groups. Reducing the cost of DAAs by 56%, 76% and 84% of the current market price would make HCV treatment cost-saving in 5, 10, and 20 years, respectively, in Japanese patients.

Previous studies on cost effectiveness of HCV treatment with DAAs have all focused on the patient cohort with one fixed age, usually 50–65 years old, according to average age of Japan’s HCV patient population^11–16^. In these studies, DAA treatment was found to be cost-effective under the current price. However, the discussion regarding the DAAs being cost-saving was lacking in related literature, and our study fills this gap by providing new data which illustrated the cost at which HCV treatment can become cost-saving in the near future. Specifically, our study considered shorter time horizons primarily for budget planning analysis.

While low-cost DAAs have already been introduced in many developing countries such as India, Pakistan and Egypt^18,40,41^, the prices of these treatments remain high for high-income countries such as Japan where generic DAA medicines are not available. While competition has helped to drive down DAA prices in the public sector in many high-income countries such as the United States^42^, Japan could also benefit by negotiating price of DAAs as described by the WHO Progress Report on Access to DAAs^43^.

While DAA treatment can be cost-saving in 5–20 years in Japan, initial investment will be needed to provide timely treatment to all HCV patients. Japan can follow an innovative financing mechanism of subscription-based payment model that has been implemented recently in Australia and is being considered in the United States and United Kingdom^44^. For instance, in the United States, Louisiana and Washington states have proposed the idea of a subscription payment mechanism, where a medicine manufacturer agrees to provide the state’s Medicaid program with unlimited access to HCV treatment for a fixed amount of money^44,45^.

Our study had some limitations. First, we did not include patients with HCV genotype 3, as the prevalence is less than 1% in Japan. Second, as with most of the previous published models on HCV treatment, our analysis did not consider the re-treatment of patients who have previously failed DAAs. Some recent DAA medicines can effectively treat patients who had not responded to other DAAs, but they are not routinely available in the Japanese market. Finally, our model excluded liver-transplant as a treatment option for HCV sequalae. However, this should not detract from our conclusions since very few liver-transplants have been performed in Japan.

In conclusion, DAA-based HCV treatment can improve patient outcomes, particularly for younger patients. However, unlike in most other countries, they are not cost-saving in Japan. Reducing the cost of DAA medicines by 55–85% would make the treatment cost-saving. Such reduction in prices would benefit the Japanese society in the long run from a healthy aging and economical perspective.

## Uncited references

(Blach et al., (2017); comment>AASLD-IDSA. Recommendations for testing, managing, and treating hepatitis C.http://www.hcvguidelines.org(last accessed: April 7, 2017)10.1002/hep.^27950^; Chung et al., (2010); Okamoto, Mishiro (1994); Ikai et al., (2007); Kumada et al., (2017); Tanaka et al., (2018); comment>Minister of Health Labor and Welfare. Hepatitis Test Acceptance Status Actual Situation Report in Japan (In Japanese)https://www.mhlw.go.jp/stf/houdou/2r9852000002gd4j.html(last accessed: November 7, 2018) (2012).; AuthGrp>Holt, S. & Hepatitis CDrug News - January, 2019Available fromhttps://www.hepatitiscentral.com/news/hepatitis-c-drugs-in-2019-price-drops-and-generic-pipeline-in-place/Last accessed: 1/10/2020; McEwan et al., (2016); Kaishima et al., (2018); Igarashi et al., (2017); McEwan et al., (2016); Ward et al., (2017); Igarashi et al., (2017); Virabhak et al., (2016); Chhatwal et al., (2015); Aggarwal et al., (2017); Bennett et al., (1997); Chhatwal et al., (2013); Siebert et al., (2003); comment>World Health Organization – Viral Hepatitis Strategic Information and Modelling Reference Group: Meeting report, 14–16 June 2016, WHO headquarters, Geneva, Switzerland. Available fromhttp://www.who.int/hepatitis/publications/strategic-information-modelling-meeting/en/(last accessed: April 14, 2017). (WHO, Geneva, 2016).; Bedossa, Poynard (1996); Afdhal et al., (2014); Nelson et al., (2015); Kohli et al., (2015); Cardoso et al., (2010); comment>World Health Organization, Global health observatory data repository. 2013. Available fromhttps://www.who.int/gho/en/last accessed: May 5, 2018; Thein et al., (2008); Fattovich et al., (1997); Wolfe et al., (2010); comment>Ministry of Health Labor and Welfare in Japan. Medical insurance database. Available fromhttp://www.mhlw.go.jp/stf/seisakunitsuite/bunya/kenkou_iryou/iryouhoken/database/index.html(last accessed: August 8, 2018); comment>Ministry of Health Labour and Welfare in Japan. Statistical information and white paper. 2012. Available fromhttp://www.mhlw.go.jp/toukei/saikin/hw/kanja/11/dl/03.pdf(last accessed August 8, 2018); comment>Ministry of Health Labour and Welfare in Japan. Various Information of Medical Fee. Available fromhttp://www.iryohoken.go.jp/shinryohoshu/(last accessed: August 8, 2018).; Chong et al., (2003); Hanmer et al., (2006); Fukuda et al., (2013); AuthGrp>Hasegawa, M., Komoto, S., Shiroiwa, T. & Fukuda, T.Formal Implementation of Cost-Effectiveness Evaluations in Japan: A Unique Health Technology Assessment SystemValue in Health10.1016/j.jval.2019.10.^005^; collab>World Health OrganizationGlobal health sector strategy on viral hepatitis 2016–2021. 2016. Available fromhttp://www.who.int/hepatitis/strategy2016-2021/ghss-hep/en/(last accessed: January 3, 2017).; Chhatwal et al., (2019); El Sherbini et al., (2015); AuthGrp>Chhatwal, J. et al.Hepatitis C retreatment in the era of direct-acting antivirals: Projections in the United StatesAlimentary Pharmacology & TherapeuticsIn press (2018).; World Health Organization (2018); Trusheim et al., (2018); Sood et al., (2018); Planas et al., (2004); Davis et al., (2011); McAdam-Marx et al., (2011); Wilson et al., (2007))

## Supplementary information

Supplementary Appendix.

## References

[1] Blach S (2017). Global prevalence and genotype distribution of hepatitis C virus infection in 2015: a modelling study. Lancet Gastroenterology Hepatology.

[2] AASLD-IDSA. Recommendations for testing, managing, and treating hepatitis C., http://www.hcvguidelines.org. (last accessed: April 7, 2017)., 10.1002/hep.27950.

[3] Chung H, Ueda T, Kudo M (2010). Changing trends in hepatitis C infection over the past 50 years in Japan. Intervirology.

[4] Okamoto H, Mishiro S (1994). Genetic heterogeneity of hepatitis C virus. Intervirology.

[5] Ikai I (2007). Report of the 17th Nationwide Follow-up Survey of Primary Liver Cancer in Japan. Hepatol. Res..

[6] Kumada H (2017). The combination of elbasvir and grazoprevir for the treatment of chronic HCV infection in Japanese patients: a randomized phase II/III study. J. gastroenterology.

[7] Tanaka, A. *et al*. *The Japanese Society of Hepatology Hepatitis C Treatment Guidelines*, https://www.jsh.or.jp/files/uploads/HCV_GL_ver6.2_v1.1.pdf (2018).

[8] Minister of Health Labor and Welfare. Hepatitis Test Acceptance Status Actual Situation Report in Japan (In Japanese)., https://www.mhlw.go.jp/stf/houdou/2r9852000002gd4j.html. (last accessed: November 7, 2018) (2012).

[9] Holt, S. & Hepatitis C Drug News - January, 2019. Available from, https://www.hepatitiscentral.com/news/hepatitis-c-drugs-in-2019-price-drops-and-generic-pipeline-in-place/. (Last accessed: 1/10/2020).

[10] McEwan P (2016). The cost-effectiveness of daclatasvir-based regimens for the treatment of hepatitis C virus genotypes 1 and 4 in the UK. Eur. J. Gastroenterol. Hepatol..

[11] Kaishima T (2018). Cost-effectiveness analyses of anti-hepatitis C virus treatments using quality of life scoring among patients with chronic liver disease in Hiroshima prefecture, Japan. Hepatol. Res..

[12] Igarashi A (2017). Cost-utility analysis of sofosbuvir for the treatment of genotype 2 chronic hepatitis C in Japan. Curr. Med. Res. Opin..

[13] McEwan P (2016). Estimating the cost-effectiveness of daclatasvir plus asunaprevir in difficult to treat Japanese patients chronically infected with hepatitis C genotype 1b. Hepatol. Res..

[14] Ward T (2017). Assessing the Budget Impact and Economic Outcomes of the Introduction of Daclatasvir + Asunaprevir and Sofosbuvir/Ledipasvir for the Treatment of Chronic Hepatitis C Virus Infection in Japan. Value Health Reg. Issues.

[15] Igarashi A (2017). Cost-utility analysis of ledipasvir/sofosbuvir for the treatment of genotype 1 chronic hepatitis C in Japan. Curr. Med. Res. Opin..

[16] Virabhak S (2016). Cost-effectiveness of direct-acting antiviral regimen ombitasvir/paritaprevir/ritonavir in treatment-naive and treatment-experienced patients infected with chronic hepatitis C virus genotype 1b in Japan. J. Med. Econ..

[17] Chhatwal J, Kanwal F, Roberts MS, Dunn MA (2015). Cost-effectiveness and budget impact of hepatitis C virus treatment with sofosbuvir and ledipasvir in the United States. Ann. Intern. Med..

[18] Aggarwal R (2017). Cost-effectiveness of hepatitis C treatment using generic direct-acting antivirals available in India. PLoS One.

[19] Bennett W (1997). Estimates of the cost-effectiveness of a single course of interferon- 2b in patients with histologically mild chronic hepatitis C. Ann. Intern. Med..

[20] Chhatwal J (2013). Cost-Effectiveness of boceprevir in patients previously treated for chronic hepatitis C genotype 1 Infection in the United States. Value Health.

[21] Siebert U (2003). Cost effectiveness of peginterferon-2b plus ribavirin versus interferon -2b plus ribavirin for initial treatment of chronic hepatitis C. Gut.

[22] World Health Organization – Viral Hepatitis Strategic Information and Modelling Reference Group: Meeting report, 14–16 June 2016, WHO headquarters, Geneva, Switzerland. Available from, http://www.who.int/hepatitis/publications/strategic-information-modelling-meeting/en/ (last accessed: April 14, 2017). (WHO, Geneva, 2016).

[23] Bedossa P, Poynard T (1996). An algorithm for the grading of activity in chronic hepatitis C. Hepatology.

[24] Afdhal N (2014). Ledipasvir and sofosbuvir for untreated HCV genotype 1 infection. N. Engl. J. Med..

[25] Nelson DR (2015). All-oral 12-week treatment with daclatasvir plus sofosbuvir in patients with hepatitis C virus genotype 3 infection: ALLY-3 phase III study. Hepatology.

[26] Kohli A (2015). Ledipasvir and sofosbuvir for hepatitis C genotype 4: a proof-of-concept, single-centre, open-label phase 2a cohort study. Lancet Infect. Dis..

[27] Cardoso AC (2010). Impact of peginterferon and ribavirin therapy on hepatocellular carcinoma: incidence and survival in hepatitis C patients with advanced fibrosis. J. Hepatology.

[28] World Health Organization, Global health observatory data repository. 2013. Available from, https://www.who.int/gho/en/ (last accessed: May 5, 2018).

[29] Thein H, Yi Q, Dore G, Krahn M (2008). Estimation of stage specific fibrosis progression rates in chronic hepatitis C virus infection: A meta analysis and meta regression. Hepatology.

[30] Fattovich G (1997). Morbidity and mortality in compensated cirrhosis type C: a retrospective follow-up study of 384 patients. Gastroenterology.

[31] Wolfe R, Roys E, Merion R (2010). Trends in Organ Donation and Transplantation in the United States, 1999–2008. Am. J. Transplant..

[32] Ministry of Health Labor and Welfare in Japan. *Medical insurance database*. *Available from*, http://www.mhlw.go.jp/stf/seisakunitsuite/bunya/kenkou_iryou/iryouhoken/database/index.html (last accessed: August 8, 2018).

[33] Ministry of Health Labour and Welfare in Japan. *Statistical information and white paper*. *2012*. *Available from*, http://www.mhlw.go.jp/toukei/saikin/hw/kanja/11/dl/03.pdf *(**last accessed August 8*, *2018**)*.

[34] Ministry of Health Labour and Welfare in Japan. Various Information of Medical Fee. Available from, http://www.iryohoken.go.jp/shinryohoshu/ (last accessed: August 8, 2018).

[35] Chong CAKY (2003). Health-state utilities and quality of life in hepatitis C patients. Am. J. Gastroenterology.

[36] Hanmer J, Lawrence WF, Anderson JP, Kaplan RM, Fryback DG (2006). Report of nationally representative values for the noninstitutionalized US adult population for 7 health-related quality-of-life scores. Med. Decis. Mak..

[37] Fukuda T (2013). Guideline for economic evaluation of healthcare technologies in Japan. J. Natl Inst. Public. Health.

[38] Hasegawa Masataka, Komoto Shigekazu, Shiroiwa Takeru, Fukuda Takashi (2020). Formal Implementation of Cost-Effectiveness Evaluations in Japan: A Unique Health Technology Assessment System. Value in Health.

[39] World Health Organization. Global health sector strategy on viral hepatitis 2016–2021. 2016. Available from, http://www.who.int/hepatitis/strategy2016-2021/ghss-hep/en/ (last accessed: January 3, 2017).

[40] Chhatwal J (2019). Assessment of the Feasibility and Cost of Hepatitis C Elimination in Pakistan. JAMA Netw. Open..

[41] El Sherbini A, Mostafa S, Ali E (2015). Systematic review with meta-analysis: comparison between therapeutic regimens for paediatric chronic hepatitis C. Aliment. Pharmacol. Ther..

[42] Chhatwal, J. *et al*. Hepatitis C retreatment in the era of direct-acting antivirals: Projections in the United States. *Alimentary Pharmacology & Therapeutics* **In press** (2018). 10.1111/apt.14527 PMC5842132 29377245

[43] World Health Organization. Guidelines for the care and treatment of persons diagnosed with chronic hepatitis C virus infection. (2018). 30307724

[44] Trusheim MR, Cassidy WM, Bach PB (2018). Alternative state-level financing for hepatitis c treatment—the “netflix model”. Jama.

[45] Sood N, Ung D, Shankar A, Strom BL (2018). A Novel Strategy for Increasing Access to Treatment for Hepatitis C Virus Infection for Medicaid Beneficiaries. Ann. Intern. Med..

[46] Planas R (2004). Natural history of decompensated hepatitis C virus-related cirrhosis. A study of 200 patients. J. Hepatology.

[47] Davis KL, Mitra D, Medjedovic J, Beam C, Rustgi V (2011). Direct economic burden of chronic hepatitis C virus in a United States managed care population. J. Clin. Gastroenterology.

[48] McAdam-Marx C (2011). All-cause and incremental per patient per year cost associated with chronic hepatitis C virus and associated liver complications in the United States: A managed care perspective. J. Manag. Care Pharm..

[49] Wilson J (2007). A systematic review and economic evaluation of epoetin alfa, epoetin beta and darbepoetin alfa in anaemia associated with cancer, especially that attributable to cancer treatment. Health Technol. Assess..

