# Supplementary material for: Estimating the price at which hepatitis C treatment with direct-acting antivirals would be cost-saving in Japan
Source: Sci Rep. 2020 Mar 5;10:4089. doi: 10.1038/s41598-020-60986-4 (PMC7058050; doi:10.1038/s41598-020-60986-4)
Supplement: Supplementary file 1 — Supplementary Appendix. [file 41598_2020_60986_MOESM1_ESM.pdf]

SUPPLEMENTARY APPENDIX

Estimating the price at which hepatitis C treatment with direct-acting antivirals would be cost-saving in Japan

Yueran Zhuo, PhD,[1*] Tomoyuki Hayashi, MD, PhD,[2,3*] Qiushi Chen, PhD,[4] Rakesh Aggarwal, MD, DM,[5] Yvan Hutin, MD, PhD,[2] Jagpreet Chhatwal, PhD,[1,6]

[1] Massachusetts General Hospital Institute for Technology Assessment, Boston, MA USA
[2] World Health Organization, Geneva, Switzerland
[3] Department of Gastroenterology, Kanazawa University and WHO Collaborating Center for Chronic Hepatitis and Liver Cancer, Kanazawa, Ishikawa, Japan
[4] The Harold and Inge Marcus Department of Industrial and Manufacturing Engineering, Pennsylvania State University, University Park, PA USA
[5] Jawaharlal Institute of Postgraduate Medical Education & Research, Puducherry, India
[6] Harvard Medical School, Boston, MA USA

*Co-lead authors

Table S1. Assumptions used for baseline population distribution of various characteristics among HCV-infected persons in Japan

Parameter	Value
**Age**	60 (20–70)
**Fibrosis score** [1]	
**F0**	4%
**F1**	12%
**F2**	10%
**F3**	19%
**F4**	55%
**Sex** [1]	
**Male**	58%
**Female**	42%
**HCV genotype** [1]*	
**G1**	65%
**G2**	34%
**G4**	1%

Abbreviations: HCV, hepatitis C virus; F, METAVIR fibrosis score; G, genotype

Data for age are shown using base case assumption and range

*HCV genotypes 3, 5 and 6 were not considered because of their rarity in Japan.

Table S2. Treatment regimens used based on HCV genotype and patients' liver fibrosis stage, and the corresponding expected rates of sustained virological response (SVR), treatment discontinuation and adverse events (AEs)

HCV genotype	METAVIR fibrosis stage	Treatment regimens	Treatment duration (weeks)	SVR (%)	Treatment discontinuation rate (%)	AEs (anemia) (%)	Duration of AEs (weeks)
**G1** [2]	F0-F3	SOF + LDV	12	98.1	1	1	2
	F4	SOF + LDV	12	93.2	1	1	2
**G2** [3,4]	F0-F3	SOF + RBV	12	94.0	1	8	4
	F4	SOF + RBV	16	94.0	1	8	4
**G4** [5]	F0-F4	SOF + LDV	12	95.0	1	2	2

Abbreviations: DCV = daclatasvir, LDV = ledipasvir, RBV = ribavirin

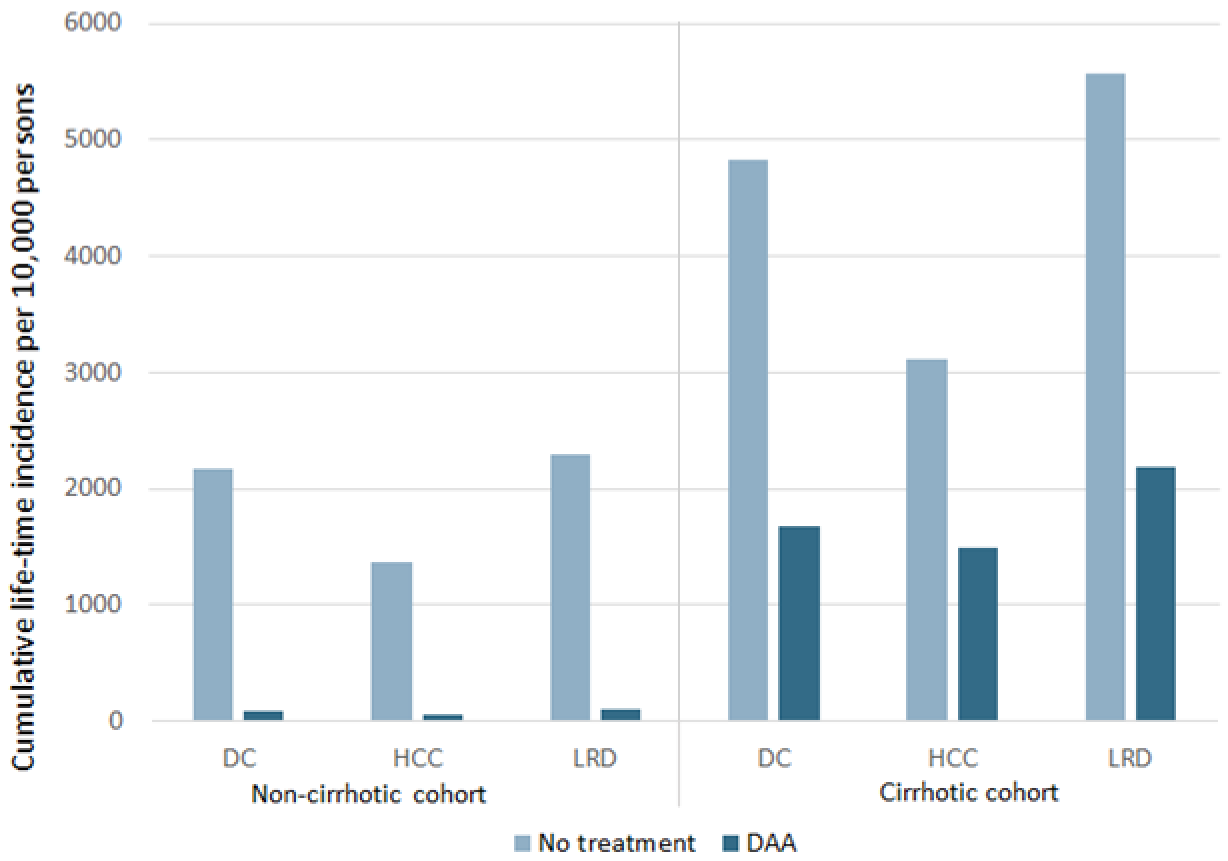

Figure S1: Change in cumulative incidence of various adverse clinical outcomes in patients with hepatitis C virus infection in Japan following treatment using regimens based on directly-acting antivirals (DAA), compared to no DAA treatment.

Abbreviations: DC, decompensated cirrhosis; HCC, hepatocellular carcinoma; LRD, liver-related death

References:

1 Blach, S. *et al.* in *Hepatology.* 372A-373A (WILEY-BLACKWELL 111 RIVER ST, HOBOKEN 07030-5774, NJ USA).

2 Afdhal, N. *et al.* Ledipasvir and sofosbuvir for untreated HCV genotype 1 infection. *N Engl J Med* **370**, 1889-1898, doi:10.1056/NEJMoa1402454 (2014).

3 Jacobson, I. M. *et al.* Sofosbuvir for hepatitis C genotype 2 or 3 in patients without treatment options. *New England Journal of Medicine* **368**, 1867-1877, doi:doi:10.1056/NEJMoa1214854 (2013).

4 Poynard, T. *et al.* Peginterferon alfa-2b and ribavirin: effective in patients with hepatitis C who failed interferon alfa/ribavirin therapy. *Gastroenterology* **136**, 1618-1628, doi:10.1053/j.gastro.2009.01.039 (2009).

5 Kohli, A. *et al.* Ledipasvir and sofosbuvir for hepatitis C genotype 4: a proof-of-concept, single-centre, open-label phase 2a cohort study. *Lancet Infect Dis* **15**, 1049-1054, doi:10.1016/S1473-3099(15)00157-7 (2015).
